# The effect of the rehabilitation program on balance, gait, physical performance and trunk rotation in Parkinson’s disease

**DOI:** 10.1007/s40520-015-0506-1

**Published:** 2015-12-10

**Authors:** Joanna Stożek, Monika Rudzińska, Urszula Pustułka-Piwnik, Andrzej Szczudlik

**Affiliations:** 1 Department of Clinical Rehabilitation, University School of Physical Education, Cracow, Poland; 2 Department of Neurology, Medical University of Silesia, Katowice, Poland; 3 Department of Neurology, Jagiellonian University Medical College, Cracow, Poland

**Keywords:** Parkinson’s disease, Rehabilitation, Motor functions, Physical performance

## Abstract

**Background:**

Parkinson’s disease (PD) is a progressive, neurodegenerative disease which leads to postural and gait disorders, limitation in mobility, activities of daily living and disability.

**Aims:**

The aim of the study is to assess the effects of the rehabilitation program on balance, gait, motor performance and trunk rotations in PD patients.

**Methods:**

Sixty-four patients with 1.5–3.0 stage PD in the Hoehn and Yahr scale were randomly allocated to rehabilitation and control groups. Sixty-one patients completed the study. Patients were assessed three times, at month intervals. Between the first and second assessments, the rehabilitation group participated in a rehabilitation training program focused on mobility, balance and gait exercises, consisting of 28 sessions. Balance was assessed with tandem stance and the Pastor test (shoulder tug). Gait was assessed with a 10 m walk at preferred speed and 360° turn. Motor performance was evaluated by means of the Physical Performance Test (PPT) and timed motor activities. The trunk rotations were measured in the lumbar and thoraco-lumbar spine with a tape measure.

**Results:**

The rehabilitation group significantly improved (*p* < 0.05) in balance and gait outcomes, PPT score, timed activities and trunk rotations both in comparison to the control group and baseline results. The positive effects of the exercise program maintained for at least 1 month.

**Conclusion:**

The 4-week rehabilitation training program focused on mobility, balance and gait exercises improved balance, gait, physical performance and trunk rotations in patients with PD.

## Introduction

Parkinson’s disease (PD) is a progressive, neurodegenerative disease which leads to limitation in mobility and activities of daily living (ADL), and in consequence to disability, dependency and decreased quality of life [1–4]. Poor functioning in daily life is associated with higher risk of falls in PD [5]. Many mobility problems in PD are associated with postural deficits and conversely: postural disturbances have their reflection in physical performance and activities of daily life [6]. Postural disturbances in PD have a wide range and can be considered in terms of postural instability, falls, difficulties in changing position of the body (e.g., sit to stand, bed mobility) [7] as well as in terms of restricted spinal, axial mobility [6, 8] and various postural deformities [9]. Schenkman et al. [6, 10] found the connection between restricted mobility of axial structures of the spine and the ability to perform activities.

Balance and gait disorders as well as bradykinesia are strongly correlated with disability in PD [1]. Furthermore, postural and gait disturbances are levodopa unresponsive symptoms [11]. According to Shulman [1], delaying and prevention of disability should be the priority of clinical management in PD. Despite optimal pharmacological treatment, disability increases in PD patients as the disease progresses [1]. Therefore, it is relevant to use rehabilitation in the treatment of PD. Although most studies indicate a beneficial influence of physiotherapy on at least some aspects of balance, gait, mobility and spinal flexibility, there are also some deficiencies in studies to date and there is a need for further, high-quality research [12].

The aim of the study is to assess the effects of the rehabilitation program on balance, gait, motor performance and trunk rotation in PD patients.

## Methods

### Participants

Participation in the study was offered to 100 consecutive PD patients attending the Movement Disorders Clinic, Department of Neurology, University Hospital in Cracow. Inclusion criteria were: diagnosis of PD according to UK PD Society Brain Bank criteria [13] established at least 6 months prior to the study, 1.5–3.0 Hoehn and Yahr stage and unchanged pharmacological treatment for at least 3 months preceding the study. The subjects’ informed written consent for participation was obtained. The study was conducted in accordance with the Declaration of Helsinki. Exclusion criteria were: severe gait disability with inability to walk unassisted, neurological, vascular or systemic disorders that may have caused permanent or intermittent weakness or instability, severe hepatic or renal insufficiency, cancer, a history of orthopedic hip or knee surgery which led to gait difficulties, other chronic disorders of the musculoskeletal system leading to restricted mobility, as well as all other contraindications to exercise.

### Measurements

The assessment of balance, gait, motor functions and trunk rotation in both rehabilitation and control groups was preceded by clinical evaluation by a neurologist with expertise on the subject of movement disorders, including a demographic and medical questionnaire and neurological examination. The severity of the disease was assessed using the Unified Parkinson’s Disease Rating Scale (UPDRS) part 3, the Hoehn and Yahr scale and the Schwab and England scale. Drug treatment was kept unchanged throughout the study.

Patients were randomly allocated into two groups: rehabilitation or control, using a random number computer generator. Patients were assessed three times at month intervals, during “on” state. Between the first and second assessments, the rehabilitation group participated in a 1-month rehabilitation program, consisting of 28 therapeutic sessions. Participants in the control group received only medication therapy. After the study was completed, two kinds of rehabilitation programs were offered to patients from the control group.

Balance was assessed with the Pastor test (shoulder tug) and tandem stance. Gait was assessed with a 10 m walk at preferred speed and 360° turn. Motor performance was assessed by means of the Physical Performance Test (PPT) and timed motor activities. The range of spinal rotation was measured in the lumbar and thoraco-lumbar spine with a tape measure. A digital stopwatch was used to time the motor tasks.

### Balance tests

#### Tandem stance

The time of maintaining balance in tandem stance was measured for a maximum of 30 s [14, 15].

#### Pastor test

Postural reactions in response to external perturbation (shoulder tug) were scored using the 5-point scale. The higher the score, the worse the balance in response to external perturbation. One point means that a subject maintains upright without taking a step and 5 points are given when a subject falls without attempting to step [14, 17].

### Gait assessment

Patients were asked to walk 10 m at a normal, preferred speed. Time and number of steps were measured, and the average step length was calculated [15, 16, 18].

The number of steps during the 360° turn was counted [16].

### Physical performance

The nine-item PPT assesses physical functional capabilities. The following maneuvers simulating daily activities were assessed: writing a sentence, simulation of eating, rising up and putting a heavy book on a shelf, dressing and taking off a jacket, picking up a coin from the floor, turning 360°, gait test, climbing stairs, number of flights during climbing the stairs (maximum 4). Seven of the nine tasks were timed and the scores for time intervals of each task were given, from 0 if task was unable to be performed to 4 if it was performed at its possible best. During the 360° turn, stability and continuity of turning were assessed. The maximum score for the nine items is 36 points [19, 20].

To assess basic motor performance, the time of the following functional tasks was measured with a stopwatch: standing up from sitting, standing up from lying on the treatment table, sitting down from standing, lying down on the treatment table from standing, lying down on the treatment table from sitting, lying down on the exercise mat from standing, rolling from supine to side lying on the treatment table, rolling from supine to prone lying on the exercise mat and standing up from lying on the exercise mat [15, 16].

### Spinal axial rotation

The range of trunk rotation was measured with a tape measure according to the Pavelka method [21]. The difference between starting and final position after maximum rotation of the trunk is the result.

The patient was seated on a chair with the feet fastened to chair legs for stability of the pelvis. The trunk rotation was measured in the lumbar (1) and thoraco-lumbar, (2) spine twice, to the right and left. The final result was an average of the two consecutive measurements.

1. The range of rotation in the lumbar spine was determined by measuring the distance between the spinous process of the fifth lumbar vertebra and xiphoid process of the sternum and after maximum rotation. In healthy people after maximum movement, the distance increases by an average 6 cm.
2. The range of rotation in the thoraco-lumbar spine was determined by measurement of the distance between the spinous process of the fifth lumbar vertebra and jugular incisure of the sternum and after maximum rotation. In healthy people after maximum movement, the distance increases by an average of 7 cm [21].

### Rehabilitation program

The rehabilitation program lasted for 4 weeks and consisted of 28 therapy sessions. Each of them lasted 2 h with breaks, two times per day during the first 2 weeks (11 therapeutic sessions, one session took place on Saturday), and during two consecutive weeks: three times per week, one session per day. Intervention was conducted in the small groups consisting of 2–3 patients.

Treatment was focused on various exercises improving balance, postural stability, walking and performance of ADL, including changing position of the body. The rehabilitation program consists of: relaxation exercises, respiratory (breathing) exercises, range of motion and stretching exercises, exercises of trunk rotation in various body positions, mobility exercises and functional training, postural re-education, balance exercises, gait training, music and elements of dance, speech therapy and exercises of facial expression as well as education (Table 1). The number of repetitions depended on the individual capacity of each patient; however, in the beginning the number was small and gradually increased as the patients’ ability improved.

**Table 1:** Examples of selected exercises

Group of exercises	Examples of exercises
Relaxation	Lying supine, listening to music, rotation of lower limbs, rolling the head on a mat from side to side
Respiratory (breathing)	Performed with arm and trunk movements, often during breaks between other exercises
Range of motion, stretching	Exercises in various positions to maintain or increase range of movement and muscle length
Mobility exercises, functional training	Trunk mobility exercises, trunk rotations, changing body position: standing up from sitting position, bed mobility, cognitive movement strategy
Postural re-education	Correction of body posture before each exercise. Learning: to correct posture, to consciously maintain upright posture, to “feel” the posture
Balance exercises	Weight-shifting exercises on various surfaces, with different feet position, postural reflex re-education
Gait training	Distance walking (with control of step length, upright posture, arm swing, etc.), “functional” walking, side and backward walking, turning, changing direction, “stop and go” exercises, on the obstacle course: avoiding, slalom between or overcoming obstacles
Dance-based exercises, simple dances and steps	Walking to various kinds of music, simple steps of dance aerobics, samba, polonaise (Polish folk dance), waltz, slow waltz, tango
Speech therapy and facial expression exercises	Sitting position, in front of a mirror: exercising voice power, articulation and facial expressions
Education	Ways of safe ADL performance, the program of systematic walking, home exercise program, active recreation, ways of overcoming freezing of gait, fall prevention

All of the exercises were performed with sensory enhancement in the form of external sensory cues, such as verbal, auditory, visual, proprioceptive or tactile stimulation. To provide sensory reinforcement and help increase the patients’ awareness of movement, we used verbal commands, counting, clapping, music, metronome, mirrors and floor markings. Exercises were performed in various body positions. Patients practiced weight-shifting exercises on various surfaces and with different feet positions. For gait training, visual cues (white, transverse lines or wooden sticks were placed at individual step lengths) as well as auditory rhythmical cues were used. Gait patterns used for both distance walking and walking during functional activities were practiced. Varying conditions: obstacles, narrow passages and places determined for turning were used. “Stop and go” exercises, changes in direction and changes in movement patterns were stressed. Patients were trained to walk during simulation of everyday life events (for instance, opening and closing doors, avoiding, slalom between obstacles or overcoming obstacles). The “attentional strategy” with verbal cues was also used to facilitate walking. Patients practiced dance-based exercises and simple dances with the aim of improving balance, initiation of movement, changing direction, trunk rotation and coordination. These types of exercises had additional motivational and social benefits.

### Data analysis

Descriptive statistics are shown as mean ± SD. The comparisons between the groups were carried out with the non-parametric Mann–Whitney test. The three consecutive assessments were compared to each other both in the rehabilitation and control groups. For this type of comparison we used Friedman’s non-parametric analysis of variance for dependent tests, and in the consecutive stage of analysis, multiple comparisons of the Duncan’s test. A *p* value <0.05 was considered statistically significant. The quantitative variables such as sex were compared with the *χ* ^2^ independence test.

## Results

Sixty-four patients with PD agreed to participate in the study and met the inclusion criteria. Sixty-one patients completed the study and their results were analyzed. One patient from the rehabilitation group, due to family issues and two patients from the control group because of unrelated medical issues and lack of time resigned from the study during its course. Finally, the two groups of patients were as follows: rehabilitation group (*n* = 30) and control group (*n* = 31). The rehabilitation and control groups did not significantly differ at baseline in demographic and clinical parameters: age, duration and severity of disease assessed with scales: Hoehn and Yahr, Schwab and England and UPDRS, part 3 (Table 2). Patients in the rehabilitation group did not significantly differ from the control group in the amount of anti-Parkinson medication.

**Table 2:** Comparison of rehabilitation and control groups

Demographic and clinical parameters	Rehabilitation group (*n* = 30)	Control group (*n* = 31)
Age (years)	64.0 ± 9.9	67.0 ± 11.3
Men/women (%)	13 (43.3)/17 (56.7)	16 (51.6)/15 (48.4)
Disease duration (years)	4.6 ± 2.7	4.3 ± 2.6
Hoehn and Yahr scale (score)	2.3 ± 0.6	2.3 ± 0.6
Schwab and England scale (score)	82.0 ± 7.1	81.6 ± 8.6
UPDRS, part 3 (score)	19.7 ± 7.8	23.2 ± 10.5

Values are mean ± standard deviation

*UPDRS* Unified Parkinson’s Disease Rating Scale

There were no statistical differences between the groups before the rehabilitation program in results of tandem stance, Pastor test, 10 m walk, 360° turn, PPT score, timed activities and trunk rotation. Comparison of balance and gait test outcomes, PPT score, timed activities and trunk rotation between the groups revealed significant (*p* ≤ 0.05) improvement in the rehabilitation group in all analyzed parameters both directly after physical therapy and after a 1-month follow-up (Table 3).

**Table 3:** Comparison of the results of balance, gait, motor performance tests and trunk rotations between groups

Test, parameter	Assessment	Group		*p*
		Rehabilitation (*n* = 30)	Control (*n* = 31)	
Pastor test (score) (shoulder tug)	1	3.10 ± 0.80	3.50 ± 0.60	NS
	2	2.30 ± 0.90	3.50 ± 0.60	0.001
	3	2.40 ± 0.90	3.50 ± 0.70	0.001
Tandem stance (s)	1	24.26 ± 10.33	20.23 ± 10.48	NS
	2	27.95 ± 5.48	19.42 ± 10.62	0.001
	3	28.29 ± 5.39	18.98 ± 10.14	0.001
10 m walk, time (s)	1	10.52 ± 4.50	13.11 ± 5.99	NS
	2	8.29 ± 1.62	12.53 ± 6.75	0.002
	3	8.43 ± 1.55	12.67 ± 4.85	0.001
10 m walk, number of steps	1	17.83 ± 3.45	19.77 ± 5.89	NS
	2	14.87 ± 2.21	18.97 ± 7.24	0.005
	3	14.83 ± 2.12	19.81 ± 5.81	0.001
10 m walk, length of step (m)	1	0.58 ± 0.10	0.54 ± 0.11	NS
	2	0.69 ± 0.10	0.57 ± 0.12	0.001
	3	0.69 ± 0.10	0.54 ± 0.11	0.001
360° turn, number of steps	1	10.70 ± 5.80	10.60 ± 6.80	NS
	2	6.90 ± 2.10	9.80 ± 4.10	0.001
	3	6.70 ± 2.00	10.80 ± 6.90	0.003
PPT (score)	1	25.00 ± 4.30	23.70 ± 4.70	NS
	2	32.20 ± 1.90	23.00 ± 4.70	0.001
	3	32.20 ± 2.00	22.90 ± 4.10	0.001
Standing up from sitting position (s)	1	1.45 ± 1.22	1.78 ± 1.29	NS
	2	0.87 ± 0.29	2.09 ± 2.63	0.001
	3	0.87 ± 0.24	1.89 ± 1.65	0.001
Standing up from lying position on treatment table (s)	1	4.89 ± 3.01	5.86 ± 3.81	NS
	2	2.86 ± 1.28	5.76 ± 3.62	0.001
	3	2.62 ± 0.96	6.09 ± 3.65	0.001
Standing up from lying position on mat (s)	1	10.01 ± 5.83	13.91 ± 9.80	NS
	2	5.63 ± 2.63	12.77 ± 10.34	0.001
	3	5.33 ± 2.61	13.38 ± 10.24	0.001
Standing up from lying to sitting position (s)	1	4.82 ± 3.85	5.38 ± 4.64	NS
	2	2.32 ± 1.05	5.38 ± 5.12	0.001
	3	2.27 ± 0.84	5.88 ± 4.95	0.001
Sitting down from standing position (s)	1	1.42 ± 0.93	1.67 ± 0.82	NS
	2	0.81 ± 0.31	1.64 ± 0.86	0.001
	3	0.88 ± 0.29	1.64 ± 0.97	0.001
Lying down on treatment table, from standing position (s)	1	5.89 ± 2.91	7.51 ± 4.63	NS
	2	3.11 ± 1.25	8.14 ± 6.04	0.001
	3	3.03 ± 1.01	7.44 ± 4.01	0.001
Lying down on treatment table from sitting position (s)	1	4.08 ± 2.48	4.65 ± 2.51	NS
	2	2.32 ± 0.85	4.95 ± 2.65	0.001
	3	2.38 ± 1.05	4.72 ± 2.21	0.001
Lying down on mat from standing position (s)	1	6.21 ± 2.81	7.98 ± 5.16	NS
	2	3.68 ± 1.47	8.73 ± 5.89	0.001
	3	3.83 ± 1.80	9.02 ± 6.52	0.001
Supine to side lying position on treatment table (s)	1	6.29 ± 5.17	6.71 ± 4.63	NS
	2	2.20 ± 0.94	6.43 ± 4.74	0.001
	3	2.65 ± 2.41	6.90 ± 4.34	0.001
Supine to prone lying position on exercise mat (s)	1	5.15 ± 3.49	6.10 ± 4.62	NS
	2	2.61 ± 1.07	7.43 ± 5.84	0.001
	3	2.71 ± 1.11	7.42 ± 4.85	0.001
Lumbar spine rotation R (cm)	1	2.96 ± 1.24	2.61 ± 1.32	NS
	2	5.07 ± 1.22	2.36 ± 1.20	0.001
	3	4.92 ± 1.18	2.44 ± 1.38	0.001
Lumbar spine rotation L (cm)	1	3.30 ± 1.40	3.07 ± 1.66	NS
	2	5.51 ± 1.41	2.86 ± 1.63	0.001
	3	5.35 ± 1.56	2.64 ± 1.53	0.001
Thoraco-lumbar spine rotation R (cm)	1	4.29 ± 2.07	4.39 ± 1.77	NS
	2	7.23 ± 1.92	4.24 ± 1.75	0.001
	3	7.21 ± 1.97	3.99 ± 1.84	0.001
Thoraco-lumbar spine rotation L (cm)	1	4.70 ± 1.98	4.76 ± 2.22	NS
	2	7.63 ± 1.84	4.46 ± 2.10	0.001
	3	7.31 ± 1.68	4.10 ± 1.98	0.001

The *p* values refer to the differences between groups in three assessments: before, after and 1 month after completing rehabilitation

Values are mean ± standard deviation

*NS* non-significant, *PPT* Physical Performance Test, *L* to the left, *R* to the right

There were no statistical differences in any parameters (balance and gait tests, PPT, timed activities, ROM in trunk rotations) of the control group during the three consecutive assessments.

There was significant improvement within the rehabilitation group of all parameters in assessments directly after and 1 month following the rehabilitation program (Table 4).

**Table 4:** Comparison of the parameters in three consecutive assessments in the rehabilitation group

Test, parameter	Assessment 1	Assessment 2	Assessment 3	*p*
Pastor test (score) (shoulder tug)	3.10 ± 0.80	2.30 ± 0.90	2.40 ± 0.90	0.001*
Tandem stance (s)	24.26 ± 10.33	27.95 ± 5.48	28.29 ± 5.39	0.003*
10 m walk, time (s)	10.52 ± 4.50	8.29 ± 1.62	8.43 ± 1.52	0.001*
10 m walk number of steps	17.83 ± 3.45	14.87 ± 2.21	14.83 ± 2.12	0.001*
10 m walk step length (m)	0.58 ± 0.10	0.69 ± 0.10	0.69 ± 0.10	0.001*
360° turn number of steps	10.70 ± 5.80	6.9 ± 2.10	6.7 ± 2.00	0.001*
PPT (score)	25.00 ± 4.30	32.2 ± 1.90	32.2 ± 2.00	0.001*
Standing up from sitting position (s)	1.45 ± 1.22	0.87 ± 0.29	0.87 ± 0.24	0.001*
Standing up from lying position on treatment table (s)	4.89 ± 3.01	2.86 ± 1.28	2.62 ± 0.96	0.001*
Standing up from lying position on mat (s)	10.01 ± 5.83	5.63 ± 2.63	5.33 ± 2.61	0.001*
Standing up from lying to sitting position (s)	4.82 ± 3.85	2.32 ± 1.05	2.27 ± 0.84	0.001*
Sitting down from standing position (s)	1.42 ± 0.93	0.81 ± 0.31	0.88 ± 0.29	0.001*
Lying down on treatment table, from standing position (s)	5.89 ± 2.91	3.11 ± 1.25	3.03 ± 1.01	0.001*
Lying down on treatment table, from sitting (s)	4.08 ± 2.48	2.32 ± 0.85	2.38 ± 1.05	0.001*
Lying down on mat, from standing position (s)	6.21 ± 8.81	3.68 ± 1.47	3.83 ± 1.8	0.001*
Supine to side lying position on treatment table (s)	6.29 ± 5.17	2.2 ± 0.94	2.65 ± 2.41	0.001*
Rolling from supine to prone lying on exercise mat (s)	5.15 ± 3.49	2.61 ± 1.07	2.71 ± 1.11	0.001*
Lumbar spine rotation R (cm)	2.96 ± 1.24	5.07 ± 1.22	4.92 ± 1.18	0.001*
Lumbar spine rotation L (cm)	3.3 ± 1.4	5.51 ± 1.41	5.35 ± 1.56	0.001*
Thoraco-lumbar spine rotation R (cm)	4.29 ± 2.07	7.23 ± 1.92	7.21 ± 1.97	0.001*
Thoraco-lumbar spine rotation L (cm)	4.7 ± 1.98	7.63 ± 1.84	7.31 ± 1.68	0.001*

Values are mean ± standard deviation

*NS* non-significant, *PPT* Physical Performance Test, *L* to the left, *R* to the right

* The *p* values refer to the difference between the first (before rehabilitation) and second (directly after rehabilitation) assessments

## Discussion

Our study shows that the rehabilitation program focused on mobility, balance and gait improved motor functions in terms of analyzed balance and gait parameters, PPT score, timed activities as well as range of trunk rotations in patients with PD. After the mobility, balance and gait physical training all parameters within the rehabilitation group significantly improved both in comparison to the control group and to baseline outcomes. The positive effect maintained at least 1 month after concluding the training program.

Our results are in agreement with the majority of other studies concerning the influence of rehabilitation on motor functions.

### Balance and gait

In our study, the steady standing in tandem position and reaction to external perturbation improved in patients after rehabilitation. Our results are consistent with the results of Morris et al. [22]. They obtained improvement in shoulder tug after only 2 weeks of physiotherapy. Morris et al. [22] compared two types of 2-week physiotherapy program: movement strategy training with musculoskeletal exercises. The movement strategy training group, but not musculoskeletal exercise group, improved after physiotherapy in the results of the shoulder tug and 10 m walk and this improvement maintained for 3 months. Ebersbach et al. [23] also obtained positive effects of two physiotherapy programs on the results of the pull test (shoulder tug) and 10 m walk. Improvement was observed after either whole body vibration training or conventional physiotherapy.

In contrast to our results, Tamir et al. [24] did not observe significant improvement in tandem stance and shoulder tug after 6 weeks of both: motor imagery practice combined with physical practice or physical practice alone. Experimental and control groups practiced twice a week for 1 h.

In our study, time, number of steps, average step length in the 10 m walk as well as the number of steps during the 360° turn improved after rehabilitation. In most studies parameters of gait improved after various kinds of exercise training programs [22, 23, 25–33]. Meta-analysis of to date studies indicates clinically significant improvement in gait speed, 2 or 6 min. walk test and Freezing of Gait Questionnaire but not in the 10 or 20 m walk test [12].

Results contrary to ours were obtained by Vivas et al. [34], after two types of physical therapy: 4-week aquatic therapy and land-based therapy. Therapies were conducted twice a week for 45 min and after completion; there was no improvement in walking speed or step amplitude. The results obtained by Schenkman et al. [35] are partially in agreement with this study. They assessed a 10-week individualized exercise program in patients with PD. After the first, randomized and controlled part of the study, the authors found significant improvement in the Functional Reach Test (FRT), Functional Axial Rotation (FAR) and the number of steps during the 360° turn. They did not report improvement in: timed 10 m walk, timed 360° turn, distance in 6 min. walk as well as in timed standing up from lying.

In our study, the number of steps during the 360° turn decreased, but in contrast to the study by Schenkman et al., the time of the 10 m walk as well as standing up from supine significantly shortened. After the second part of physiotherapy, the authors compared the baseline and final results of all patients from the experimental (23) and control (22) groups, and observed improvement in FRT, FAR, time and number of steps in 360°, time of standing up from lying, but not in the time of the 10 m walk, distance of 6 min walk and timed lying down.

The results of Schenkman et al. [35] show, similarly to our results, improvement in number of steps during the 360° turn but also in contrast, lack of positive changes in the 10 m walk.

### Physical performance

In our study, it was indicated that after rehabilitation, the time of all performed activities shortened in patients with PD. Performance of the following activities improved: sit to stand, rising from lying to sitting and standing, lying down, rolling from supine to prone and side lying, and the score of the PPT increased significantly. All of the parameters improved significantly in the rehabilitation group both in comparison to the control group and to baseline assessment.

Despite optimized pharmacological treatment, PD leads to progressive loss of functional mobility of patients and decreases the possibility of leading an independent life. Much et al. [36] found that a high percentage of patients, in addition to other motor problems, such as: walking, dressing, movement initiation; suffer from difficulty in performing ADL, including rolling over in bed (67.2 %) and standing up from sitting (69.8 %). Only a few studies concern the influence of physical therapy on specific motor dysfunctions, impairing simple, everyday activities such as standing up from sitting position, rolling over in bed and getting out from bed for motor assessment.

The results of our study are in agreement with the study of Smania et al. [37]. They studied the effect of balance training in Parkinson’s disease patients. Twenty-eight patients underwent balance training which consisted of 21 treatment sessions and the control group received general exercises for performance. Patients were assessed among other measures with the postural transfer test: timed lying to sitting and sitting to standing. After therapy, the time of transfers significantly improved in the intervention group and maintained for at least 1 month.

Similar to our results, Villani et al. [38] obtained a shortening in the time of performed activities. 20 patients with mild to moderate PD participated in a 5-week physical therapy program, twice a week, in groups of 6–7 patients. Participants significantly improved their time of: rising from lying to sitting, lying down from sitting to lying, rolling from supine to side lying and standing up from sitting. There was no control group in their study.

### Trunk rotations

In our study, trunk rotations in lumbar and thoracolumbar spine significantly increased after four-week rehabilitation in patients with PD. Similarly to our study, Schenkman et al. [35] obtained improvement in functional axial rotation (FAR) after rehabilitation. Bartolo et al. [8] observed a significant increase in range of trunk flexion and lateral bending after 4-week rehabilitation. They also found improvement in posture and a decrease in trunk lateral flexion and inclination in standing position in patients with PD. A consequence of PD is loss of flexibility and altered posture. The authors have suggested that lack of spinal flexibility may contribute to difficulty with balance control and physical limitations for people with PD [6, 8, 9, 35, 39]. Trunk rotation contributes to many postural activities, such as: rolling over, walking, turning during walking. According to Stieger et al. [40], impairment of axial movement is a common cause of disability in PD patients. Similar to other studies of the trunk range of motion in PD [6, 8, 35], we have observed that trunk rotation is restricted in PD patients but can increase after rehabilitation.

Our study and most of the other recent studies concerning the influence of physical training on various motor functions in PD indicate improvement in balance, gait, mobility and spinal flexibility after rehabilitation. Balance improved both in ability to maintain relatively difficult tandem stance and in reaction to external perturbation. Gait improved in time, amplitude of steps, number of steps in 10 m walk and the number of steps during the 360° turn. Time and quality of physical performance as well as trunk rotations improved in exercising patients with PD after rehabilitation.

The limitation of this study is the short observation period of the effects. Further studies are needed to optimize physiotherapy aimed at preventing disability in Parkinson’s disease. Following studies should determine the sufficient and effective dose of physical exercises in a wide range of possibilities and forms of physical training and therapeutic activities.

## Conclusion

Our study indicates that balance and gait disorders as well as poor physical performance which lead to immobility and disability in PD can improve after rehabilitation. The positive effects noted in our study support the need for rehabilitation in PD. The 4-week rehabilitation training program focusing on mobility, balance and gait exercises indicated improvement in balance, gait, physical performance and trunk rotation in patients with PD.
